# Blocking c-MET/ERBB1 Axis Prevents Brain Metastasis in ERBB2+ Breast Cancer

**DOI:** 10.3390/cancers12102838

**Published:** 2020-10-01

**Authors:** Shailendra K. Gautam, Ranjana K. Kanchan, Jawed A. Siddiqui, Shailendra K. Maurya, Sanchita Rauth, Naveenkumar Perumal, Pranita Atri, Ramakanth C. Venkata, Kavita Mallya, Sameer Mirza, Moorthy P. Ponnusamy, Vimla Band, Sidharth Mahapatra, Maneesh Jain, Surinder K. Batra, Mohd Wasim Nasser

**Affiliations:** 1 Department of Biochemistry and Molecular Biology, University of Nebraska Medical Center, Omaha, NE 68198, USA; shailendra.gautam@unmc.edu (S.K.G.); ranjana.kanchan@unmc.edu (R.K.K.); jawed.siddiqui@unmc.edu (J.A.S.); shailendra.maurya@unmc.edu (S.K.M.); sanchita.rauth@unmc.edu (S.R.); naveenkumar.perumal@unmc.edu (N.P.); p.atri@unmc.edu (P.A.); r.chirravurivenkata@unmc.edu (R.C.V.); kmallya@unmc.edu (K.M.); mpalanim@unmc.edu (M.P.P.); vband@unmc.edu (V.B.); sidharth.mahapatra@unmc.edu (S.M.); mjain@unmc.edu (M.J.); sbatra@unmc.edu (S.K.B.); 2 Department of Genetics, Cell Biology and Anatomy, University of Nebraska Medical Center, Omaha, NE 68198, USA; smirza@unmc.edu; 3 Fred and Pamela Buffett Cancer Center, University of Nebraska Medical Center, Omaha, NE 68198, USA; 4 Department of Pediatrics, University of Nebraska Medical Center, Omaha, NE 68198, USA

**Keywords:** breast cancer, brain metastasis, targeted therapy, c-MET/ERBB1 axis, combination therapy, orthotopic model, spontaneous metastasis, organoids, neratinib, cabozantinib

## Abstract

**Simple Summary:**

Targeted monotherapies are ineffective in the treatment of brain metastasis of ERBB2^+^ breast cancer (BC) underscoring the need for combination therapies. The lack of robust preclinical models has further hampered the assessment of treatment modalities. We report here a clinically relevant orthotopic mouse model of ERBB2^+^ BC that spontaneously metastasizes to brain and demonstrates that targeting the c-MET/ERBB1 axis with a combination of cabozantinib and neratinib decreases primary tumor growth and prevents brain metastasis in ERBB2^+^ BC.

**Abstract:**

Brain metastasis (BrM) remains a significant cause of cancer-related mortality in epidermal growth factor receptor 2-positive (ERBB2^+^) breast cancer (BC) patients. We proposed here that a combination treatment of irreversible tyrosine kinase inhibitor neratinib (NER) and the c-MET inhibitor cabozantinib (CBZ) could prevent brain metastasis. To address this, we first tested the combination treatment of NER and CBZ in the brain-seeking ERBB2^+^ cell lines SKBrM3 and JIMT-1-BR3, and in ERBB2^+^ organoids that expressed the c-MET/ERBB1 axis. Next, we developed and characterized an orthotopic mouse model of spontaneous BrM and evaluated the therapeutic effect of CBZ and NER in vivo. The combination treatment of NER and CBZ significantly inhibited proliferation and migration in ERBB2^+^ cell lines and reduced the organoid growth in vitro. Mechanistically, the combination treatment of NER and CBZ substantially inhibited ERK activation downstream of the c-MET/ERBB1 axis. Orthotopically implanted SKBrM3^+^ cells formed primary tumor in the mammary fat pad and spontaneously metastasized to the brain and other distant organs. Combination treatment with NER and CBZ inhibited primary tumor growth and predominantly prevented BrM. In conclusion, the orthotopic model of spontaneous BrM is clinically relevant, and the combination therapy of NER and CBZ might be a useful approach to prevent BrM in BC.

## 1. Introduction

The improved five-year survival rate of ~90% of breast cancer (BC) patients is mainly attributed to its successful clinical management that includes early screening and effective treatment modalities [1]. However, for BC patients with distant metastasis, the five-year survival rate is ~27%, with a median survival of 18–24 months [2]. Irrespective of molecular subtypes, BC patients with brain metastasis (BrM) have the worst cancer-specific survival (CSS). Retrospective analysis of the Surveillance, Epidemiology, and End Results (SEER) database of BC patients indicates that the ERBB2^+^ subtype along with triple-negative BC account for more than 50% of cases of distant metastasis, which preferentially metastasizes to brain, bone, liver, and lungs [3]. Despite available targeted therapies, metastatic ERBB2^+^ BC patients have a median survival of ~34 months [3]. The poor therapeutic response towards targeted therapies in metastatic ERBB2^+^ BC is attributed in part to clonal evolution in metastatic cells, their adaptation to the organ-specific microenvironment, and differential drug delivery to the metastatic niche [4,5,6]. This is exemplified by the presence of the blood–brain barrier (BBB) in the case of BrM, which has one of the poorest outcomes among all metastatic BCs [7,8,9]. 

The limited success of available therapeutic approaches against metastatic ERBB2^+^ BC underscores the need for novel targeted therapies. Recent clinical studies with small molecule inhibitors, including tyrosine kinase inhibitors, anti-ERBB2 agents, PI3K/Akt/mTOR inhibitors, and CDK4/6 inhibitors, have shown promise in inhibiting proliferation and metastasis in ERBB2^+^ BC [10,11,12]. For instance, inhibitors targeting ERBB1 and c-MET receptors that are upregulated during BC metastasis, are currently being investigated in clinical trials for patients with existing metastasis [13,14,15,16]. Neratinib (NER), an irreversible pan-ERBB family inhibitor, has been reported to be efficacious in metastatic BC patients in combination with capecitabine [17] and paclitaxel [18,19]. Further, the data from two different clinical trials showed that NER, in combination with either capecitabine or T-DM1, was effective against brain metastatic ERBB2^+^ BC, with grade 3 and 4 levels of toxicity, respectively [14,15]. A recent study in the ERBB2^+^ spontaneous metastasis model showed that NER monotherapy (60 mg/kg) inhibited proliferation and distant metastasis in BALB/c mice via inhibition of ferroptosis [20]. In addition, fluorescent imaging at experimental end-point post-neoadjuvant treatment with NER showed preventive effects on metastatic progression. Similarly, cabozantinib (CBZ), an inhibitor of the c-MET receptor, alone or in combination with standard therapies, is being investigated in ongoing clinical trials for the treatment of patients with metastatic breast and renal cell carcinomas [13,21,22]. As both ERBB1 and c-MET have been reported to be upregulated in metastatic ERBB2^+^ BC and positively correlated with poor survival in ERBB2^+^ BC patients, these pathways are considered as potential targets for combination therapies of metastatic BC. However, the effect of combination therapy, targeting both ERBB1 and c-MET has not been investigated in brain metastatic ERBB2^+^ BC.

There are limited preclinical models available to understand underlying mechanism and evaluate targeted therapies against BrM of ERBB2^+^ BC. So far, mostly intracardiac and intracarotid injection-based models have been used to investigate therapeutic approaches against BC BrM [23,24,25]; however, both intracardiac and intracarotid models are considered insufficient to represent BC pathogenesis. In particular, the lack of primary tumors in these models limits the therapeutic evaluation of different treatment modalities to the metastatic site only, which is not the case in clinical management of metastatic BC (MBC) patients. In addition, the intracardiac and intracarotid models do not recapitulate the incidence of spontaneous metastasis and are often considered as models of “forced metastasis”. Although an orthotopic ERBB2^+^ BC model for spontaneous metastasis has been reported recently in BALB/c mouse, it showed 80% incidences of lung and adrenal metastases, respectively, and 50% and 60% metastasis to bone and brain, respectively [20]. Thus far, there is a lack of clinically relevant ERBB2^+^ mouse model that can recapitulate the incidences of distant metastasis like MBC patients. Particularly, a mouse model demonstrating spontaneous metastasis to the brain is required to evaluate therapeutic modalities against lethal BrM of BC.

In the present study, we aimed to evaluate the combined efficacy of ERBB1 and c-MET targeted therapies in vitro in brain metastatic ERBB2^+^ cell lines and in an organoid model, and in vivo in a novel orthotopic model of spontaneous BC metastasis. We observed that FDA approved anti-ErbB1/ErbB2 neratinib (NER) and anti-c-MET cabozantinib (CBZ) inhibited proliferation and metastasis of brain seeking ERBB2^+^ SKBrM3 and JIMT-1-BR3 BC cell lines and decreased growth of organoids derived from huERBB2-Tg mice. To evaluate combination therapy in vivo, we developed a unique ERBB2^+^ BC orthotopic nude mouse model of spontaneous metastasis that showed primary tumor growth and clinically relevant distant metastasis to brain, bone, liver, and lung. Combination treatment with NER and CBZ for 3 weeks was effective in inhibiting the tumor growth and incidence of BrM. Further, the combination treatment was more effective in preventing brain metastasis with a partial effect on other metastatic sites. Altogether, treatment with the NER inhibitor alone, and in combination with a CBZ, is an effective strategy for preventing BrM as observed in the orthotopic model of spontaneous BC metastasis.

## 2. Results

### 2.1. Targeting ERBB1 and c-MET Inhibits Proliferation in Brain-Seeking BC Cell Lines and ERBB2^+^ Organoids

**Figure 1:**
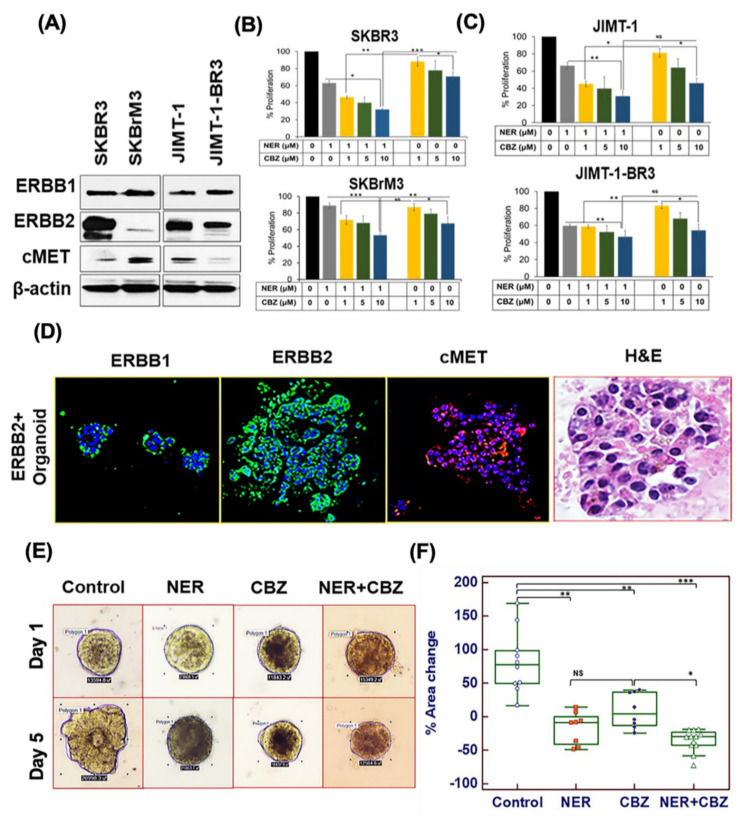
Effect of targeting ERBB1 and c-MET on cell proliferation in brain-seeking breast cancer (BC) cell lines and huHER2 tumor-derived organoids. (**A**) Expression of ERBB1, c-MET receptor, and ERBB2 in brain-seeking cell lines SKBrM3 and JIMT-1-BR3 and their respective parental cell lines SKBR3 and JIMT-1 by Western blotting. (**B**,**C**) Effect of treatment with NER, cabozantinib (CBZ), or their combination on proliferation of SKBrM3 and JIMT-1-BR3 cells as compared to their parental cell lines. The x-axis shows the treatment group for each cell line, whereas the y-axis shows percent cytotoxicity in different combination treatments in the MTT assay. Concentrations of each treatment are mentioned on X-axis. (**D**) Expression of ERBB1 (Alexa488), ERBB2 (Alexa488), and c-MET (Alexa568) receptors analyzed by immunofluorescence assay. H&E staining in the right panel shows the morphology of the organoids. The scale bar for IF images was 20 μm, whereas the scale bar for H&E of organoids in right panel was 100 μm. (**E**,**F**) Effect of treatments on organoids. Panel E shows a representative image of each group including control, NER, CBZ, and their combination (scale bar = 200 μm), whereas panel F shows quantitative data for the effect of drugs on the growth of huERBB2^+^ organoids. The area for each organoid was calculated in μm^2^, and the percent change in area of organoids (y-axis) was plotted for each treatment group (x-axis). Growth was measured in *n* = 8 organoids for NER and CBZ treatment groups and *n* = 10 and *n* = 11 organoids for control and combination groups, respectively. The statistical significance among different groups was calculated by one-way ANOVA with * *p* < 0.01; ** *p* < 0.001; and *** *p* < 0.0001; NS = No significance.

Previous reports suggest that ERBB1/2 and c-MET pathways play an important role in metastatic progression of different cancers, including BC [26,27,28,29]. We analyzed the expression of ERBB1, ERBB2, and c-MET receptors in the ERBB2^+^ brain metastatic BC cell lines SKBrM3 and JIMT-1-BR3. Interestingly, we observed an increased expression of ERBB1 in both SKBrM3 and trastuzumab-resistant JIMT-1-BR3 cell lines, but relatively reduced expression of ERBB2, as compared to their respective parental cell lines SKBR3 and JIMT-1 (Figure 1A). In contrast, c-MET expression was upregulated only in SKBrM3 cells as compared to its parental cell line (Figure 1A and Figure S1A). To investigate the efficacy of NER, we first estimated the inhibitory concentration in SKBrM3 and JIMT-1-BR3 cell lines. The IC_50_ of NER for SKBrM3 and JIMT-1-BR3 were estimated as 7.2 μM and 3.3 μM, respectively (Figure S1B). Based on the inhibitory effect in SKBrM3 cells, we treated all the cell lines with 1 μM NER (below IC_20_ of SKBrM3 cell line) alone or in combination with different concentrations of CBZ (1–10 μM). The combination of NER and CBZ significantly inhibited cell proliferation (46.71% ± 4.6%) as compared to NER alone (10.98 ± 3.2%) and CBZ alone (32.46% ± 8.1%) in SKBrM3 cells (Figure 1B), whereas in the SKBR3 cell line, the combination treatment inhibited proliferation to a greater extent (68.1% ± 1.3%) compared to NER alone (36.8 ± 2.8%) and CBZ alone (29.2 ± 5.4%), but the fold-difference in growth inhibition was lower compared to the SKBrM3 cell line (Figure 1B). These studies suggested that the combination of NER and CBZ inhibited the growth of SKBrM3 cells in a dose-dependent and synergistic manner. In contrast, we did not observe a synergistic effect of combination treatment in the JIMT-1-BR3 cell line (Figure 1C). As JIMT-1-BR3 showed lower expression of the c-MET receptor, we did not pursue it for evaluation of combination therapy targeting the c-MET/ERBB1 axis. Based on the results in the proliferation assay, we selected NER (1 μM) and CBZ (5 μM) for further treatments. 

Next, we investigated the effect of NER and CBZ on the organoids that were generated from huERBB2^+^ transgenic (Tg) mice. Here, we first analyzed the expression of targets pertinent to the combination treatment. We observed that ERBB1, ERBB2, and c-MET were highly expressed in these groups. Interestingly, compared to the 84.6% ± 22.2% change in the area of organoids in the control group (*n* = 10), the percent change in area for NER treatment was −16.72 ± 22.3% (** *p* < 0.01); for CBZ treatment 8.9 ± 24.3% (** *p* < 0.01); and for NER+CBZ treatment −43.06 ± 16.8% (** *p* < 0.01). Among the treatment groups, both NER and CBZ decreased proliferation as compared to the untreated control (Figure 1E,F). However, there was no significant difference in organoid growth between NER and CBZ treatment groups. Further, the combination treatment with NER and CBZ significantly reduced organoid growth as compared to the control group (~4-fold reduction; *** *p* < 0.001) and to single-agent treatments (Figure 1E,F). These data suggested that the combination of NER and CBZ was effective in the ERBB2^+^ organoid model and, therefore, required further investigation in an appropriate in vivo model of metastasis.

### 2.2. Effect of NER and CBZ Treatment on Migration of Brain Seeking Cells

**Figure 2:**
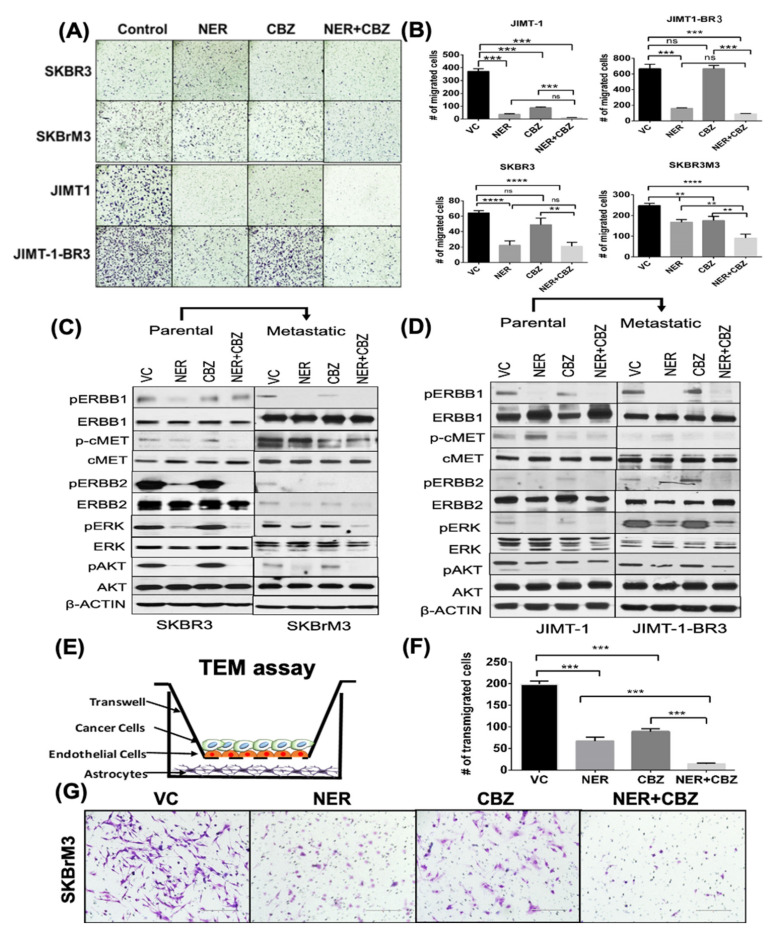
Effect of combination treatment on downstream signaling and in vitro metastatic behavior. (**A**,**B**) Effect of combination treatment on in vitro migratory potential of SKBrM3 and JIMT-1-BR3 cell lines. Brain-seeking BC cell lines SKBrM3 and JIMT-1-BR3 and their respective parental cell lines SKBR3 and JIMT-1 were treated with NER (1 μM), CBZ (5 μM), or their combination. Cells that migrated through the Boyden chamber were stained (**A**) and quantified (**B**) to analyze the effect of treatment on cell migration; scale bar = 1000 μm. (**C**,**D**) Cell lysates collected after 48 h of treatment with NER (1 μM) and CBZ (5 μM) were analyzed by Western blotting. Expression levels of target molecules pERBB1/ERBB1, pERBB2/ERBB2, and pc-MET/c-MET were analyzed along with downstream pERK/ERK and pAKT/AKT signaling molecules in SKBrM3 cells and parental cell line SKBR3 (**C**), and in JIMT-1-BR3 and parental cell line JIMT1 (**D**). Beta-actin expression was used as a loading control for each cell line. (**E**) Schematic representation of trans-endothelial migration (TEM) assay. (**F**) Trans-endothelial migration was quantified in SKBrM3 cells treated with NER and CBZ alone, or their combination. (**G**) Micrograph showing CBZ and NER treated SKBrM3 cells transmigrated in trans-endothelial assay; scale bar = 400 μm. Experiments were performed in triplicate, and a paired t-test was used to calculate the statistical significance (**** *p* < 0.0001; *** *p* < 0.005; ** *p* < 0.01; * *p* < 0.05, ns = No significance).

We performed a Boyden chamber migration assay to evaluate the effect of combination therapy on cell migration. Interestingly, we observed that NER (1 μM) and CBZ (5 μM) concentrations each inhibited in vitro cell migration of SKBrM3 as well as SKBR3 cell lines (Figure 2A). In the SKBrM3 cell line, NER and CBZ alone inhibited migration by 32.3 ± 2.9% and 29.2 ± 4%, respectively, compared to the untreated control group (Figure 2B). The effect was even greater with a combination of NER and CBZ in the SKBrM3 cell line (63.25 ± 7.6%), suggesting that targeting the ERBB1 and c-MET receptors inhibits cell motility in the SKBrM3 cell line. In contrast, CBZ alone significantly reduced the migration of JIMT-1, but not JIMT-1-BR3 cells (Figure 2A), possibly due to reduced expression of c-MET in the latter cell line. These studies suggested that the c-MET receptor might not be a potential target in JIMT-1-BR3 cells. However, NER treatment reduced the migration of JIMT-1-BR3 cells by 76 ± 1.6% (Figure 2B) as compared to the untreated control group. The quantitative analysis showed that more SKBrM3 cells migrated through the 0.8 μm barrier as compared to the parental cell line, possibly due to their higher metastatic potential (Figure 2B).

### 2.3. Effect of NER and CBZ on Downstream Signaling

The synergistic regulation of signaling mediated by ERBB1 and c-MET receptors is important in the regulation of cancer progression, metastasis, and drug resistance [16,28,30]. The downstream protein kinase B (PKB/Akt) and extracellular signal-regulated kinase (ERK) are co-regulated by both ERBB1 and c-MET receptors [31]. Therefore, we analyzed the effect of NER (1 μM) and CBZ (5 μM) for 48 h on the expression of ERBB1 and c-MET receptors, and assessed the effect of combination treatment on activation of AKT and ERK molecules in SKBrM3, JIMT-1-BR3, and their respective parental cell lines. Interestingly, NER, as a single agent, as well as in combination with CBZ, modulated pERBB2 (Tyr1248), pERBB1 (Tyr1068), and its downstream pAKT (Ser473) and pERK (Thr982) signaling (Figure 2C,D). Particularly in metastatic SKBrM3 and JIMT-1-BR3 cell lines, 1 μM NER treatment reduced downstream pERK and pAKT expression, suggesting that NER treatment alone is efficacious in inhibiting downstream signaling. In contrast, CBZ at 5 μM had no effect on ERK and AKT phosphorylation. Furthermore, a combination with NER (1 μM) and CBZ (5 μM) reduced pERK signaling with a partial effect on pAKT signaling (Figure 2C,D), suggesting that downstream pERK signaling is critical in the metastatic SKBrM3 cell line. Further, we observed a similar response with NER alone or NER in combination with CBZ on the JIMT-1-BR3 cell line that expressed a low endogenous level of c-MET, suggesting specificity of CBZ with c-MET expression.

### 2.4. Effect of NER and CBZ on In Vitro Trans-Endothelial Migration

The BBB is selectively permeable under normal physiological conditions. However, the pathological cues, including brain metastasis, render the loss of BBB integrity, which transforms the intact BBB into the blood–tumor barrier (BTB) and alters the permeability for therapeutic agents [32,33,34]. As the SKBrM3 cell line expresses high c-MET and ERBB1 receptors, and the combination treatment of NER and CBZ predominantly downregulated the pERK pathway in this cell line, we examined the effect of treatment on the migration of SKBrM3 cell line in vitro that mimics the human BBB to some extent (Figure 2E). We also observed a greater impact of combination treatment in the trans-endothelial migration (TEM) assay. Interestingly, as compared to the control group, NER and CBZ alone inhibited the TEM 66.16% ± 3.18 and 55.10% ± 2.17, respectively, whereas inhibition of migration was significantly greater in the combination treatment (92.79% ± 0.89; Figure 2F). Further, to visualize the effect of combination therapy on TEM of SKBrM3 cells, we presented the micrograph data, which showed that the combination treatment with NER and CBZ elicited a more profound effect as compared to control and single agent treatments (Figure 2G). In addition, the efficacy of combination treatment of NER and CBZ was significantly higher than single treatment groups, suggesting that targeting the c-MET/ERBB1 axis could be a potential therapeutic strategy to prevent brain metastasis. 

### 2.5. Characterization of the Orthotopic Model of Spontaneous BC Metastasis 

**Figure 3:**
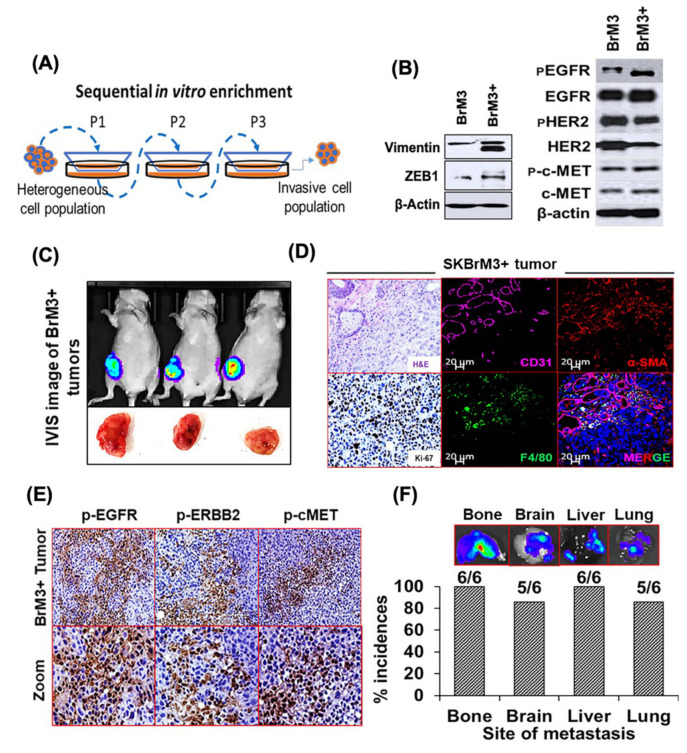
Development and characterization of an orthotopic mouse model for BC. (**A**) Schematic to show in vitro enrichment of migration potential in SKBrM3 cells by sequential passaging through a Boyden chamber (SKBrM3^+^ or BrM3^+^), as mentioned in Material and Methods. (**B**) The expression levels of pERBB1/ERBB1, pERBB2/ERBB2, pc-MET/c-MET, and the metastasis markers vimentin and ZEB1 were analyzed in SKBrM3^+^ cells using immunoblotting. (**C**,**D**) Characterization of an orthotopic model of BC metastasis in athymic nude mice. (**C**) Representative bioluminescence imaging (BLI) images and pictures of harvested orthotopic tumors and their histological and proliferation analysis stained by H&E (upper left) and Ki-67 (lower left) (scale bar = 20 μm). (**D**) IF staining of tumor sections for the analysis of macrophages (F4/80), blood vessels (CD31), and fibroblasts (α-SMA) (**E**) Expression of pERBB1, pERBB2, and pc-MET in SKBrM3^+^ tumors derived from nude mice (scale bar = 1000 μm). Images were magnified (lower panel) to highlight the tumor sections with positive staining. (**F**) Representative BLI images of isolated organs and percent incidences of distant metastasis in the SKBrM3^+^ BC orthotopic model.

Animal models developing distant metastasis either due to spontaneous progression of BC or by orthotopic implantation in mammary fat-pads are useful to understand the molecular mechanisms regulating various steps of metastatic progression and for evaluating the anti-tumor and anti-metastatic activity of targeted therapies. To develop a BC orthotopic model of spontaneous metastasis, we selected SKBrM3 cells, as these cells express high endogenous levels of c-MET and ERBB1 and showed an adequate response in combination in vitro compared to other cell lines. We first enriched SKBrM3 cells using the Boyden chamber as demonstrated in the schematic (Figure 3A). The enriched SKBrM3 cells (SKBrM3^+^ hereafter) exhibiting high vimentin and ZEB1 expression (Figure 3B) were used for orthotopic injection in the fat pad of the fourth mammary gland of female nude mice (*n* = 6). We observed a progressive tumor growth kinetics and after 6 weeks, we euthanized tumor-bearing mice to record tumor weights and incidences of metastasis. The average tumor weight was found to be 1.43 ± 0.32 g. Representative images from bioluminescence imaging (BLI) of orthotopic tumors and pictures of isolated tumors are shown with in Figure 3C. Subsequently, we assessed the composition of the tumor microenvironment in SKBrM3^+^ orthotopic tumors. The IF staining suggested that the tumors were highly positive for α-SMA (fibroblasts), CD31 (blood vessels), and F4/80 (macrophages), which are considered as major cellular constituents of the tumor microenvironment and play an instrumental role in metastatic progression [35,36,37,38] (Figure 3D). Next, we analyzed the expression of target molecules in the tumor sections derived from the mammary fat pads of implanted mice. 

Interestingly, we observed high expression of pERBB1, pERBB2, and pc-MET in SKBrM3^+^ tumor sections in Immunohistochemistry (IHC) analysis (Figure 3E). Furthermore, we isolated the organs and performed BLI to analyze incidences of metastasis in different organs. Interestingly, mice bearing orthotopic tumors exhibited extensive distant metastasis to the bone, brain, liver, and lung, which are the major organs of metastasis in the ERBB2^+^ BC subtype (Figure 3F). Representative images from BLI are shown for each organ along with the total incidences of metastasis (Figure 3F). Further, we observed that SKBrM3^+^ cells exhibited high incidences of brain metastasis, and we found five mice out of six to be positive in BLI. The organ-specific metastatic behavior of SKBrM3^+^ cells might be a cumulative result of sequential in vitro enrichment, the injection site, and/or influence of the tumor microenvironment on disseminated cells. 

### 2.6. NER and CBZ Treatment Decreases Tumor Growth and Prevents Distant Metastasis 

**Figure 4:**
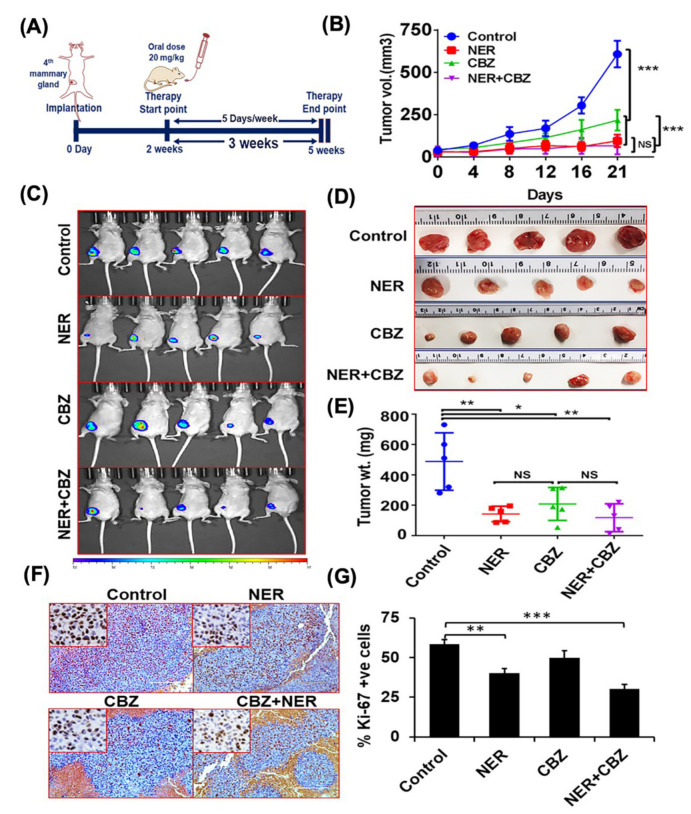
In vivo efficacy of combination treatment against tumor growth in the orthotopic model of metastasis. (**A**) Experimental plan for analyzing the efficacy of NER, CBZ, and their combination in an athymic mouse model of metastasis. (**B**) Tumor growth kinetics (*n* = 5 mice/group) after 3 weeks of therapy in different groups including NER (20 mg/kg/mouse), CBZ (20 mg/kg/mouse), and their combination. (**C**) BLI of mice from each control and treatment groups at the experimental endpoint was performed using the small animal imaging platform. Following live animal imaging, mice were sacrificed, and primary tumors were harvested as photographed (**D**), and weighed for each treatment group (**E**). (**F**,**G**) SKBrM3^+^ tumor sections were stained for Ki-67 expression in different treatment groups to analyze the effect of combination treatment on the percent proliferating cells (Scale bar = 1000 μm: upper right panel in each image showed magnified image). Statistical significance was calculated on GraphPad software using one-way ANOVA to compare tumor growth in different treatment groups with vehicle control (* *p* < 0.05; ** *p* < 0.01, *** *p* < 0.001; NS = No significance).

As the upregulated ERBB1 and c-MET pathways contribute to BC progression and distant metastasis [26,27,28], we investigated the ability of NER and CBZ to prevent primary tumor growth and metastasis in the orthotopic model of spontaneous metastasis (*n* = 5). Based on previous studies, we considered lower doses of NER and CBZ (20 mg/kg body weight each) to investigate combined efficacy in vivo [39,40]. As mentioned in the experimental plan, we administered NER and CBZ orally five days a week for three weeks (Figure 4A). Following three weeks of treatment, we analyzed the effect of combination therapy on tumor growth and metastasis. Interestingly, both NER (20 mg/kg/body weight) and CBZ (20 mg/kg/body weight) decreased the average tumor volume by 85.7% and 67.4%, respectively, as compared to the control group (Figure 4B). The combination treatment further decreased the average tumor volume by 90.2% of the control group (Figure 4B), which was also observed in BLI before euthanization of mice at experimental endpoint (Figure 4C), and in the images of isolated tumors (Figure 4D). This data was further substantiated by analysis of isolated tumors from each group, where we observed that the average tumor weights were significantly decreased in each treatment group including NER (70.8% ± 10.45%; ** *p* < 0.01), CBZ (58.25% ± 14.2%; * *p* < 0.05), and combination (75.82% ± 18.74%; ** *p* < 0.01) as compared to the control group (Figure 4E). Further immunohistochemical analysis of the primary tumors demonstrated significantly lower Ki-67^+^ cells in the combination treatment groups as compared to the control group and single treatment groups (Figure 4F,G). Overall, the combination treatment of NER and CBZ significantly reduced the tumor growth, as compared to CBZ alone and untreated control group.

**Figure 5:**
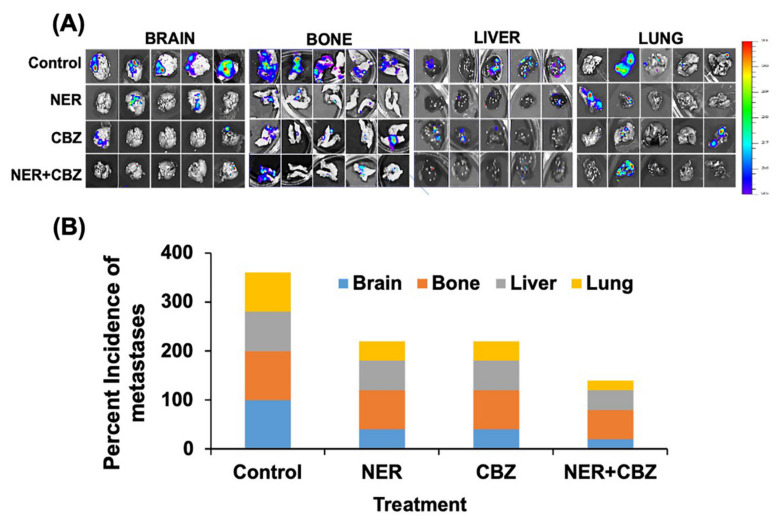
Effect of targeting the ERBB-c-MET axis on distant metastasis. (**A**) Post-therapy IVIS imaging of isolated metastatic organs including brain, bone, lung, and liver. Organs were harvested 3 weeks after the therapy with NER, CBZ, and their combination. (**B**) Stacked bar-graph to show the quantification of organ-specific metastasis using BLI, where the x-axis shows the percent incidences for metastasis in each organ including brain (blue), bone (orange), liver (gray), and lung (yellow), and the y-axis shows different treatment groups including control, NER, CBZ, and their combination.

We further analyzed the effect of combination treatment with NER and CBZ on incidences of metastasis by a using small animal imaging system. The BLI analysis of isolated organs suggested that the combination of NER and CBZ predominantly showed a preventive effect on the incidences in BrM (Figure 5A,B). We found that 80% of mice did not show incidences of BrM after the treatment with combination treatment. In contrast, the combination of NER and CBZ showed a preventive effect to a lesser extent in the case of bone metastasis, but we observed reduced metastatic burden in bone post treatment, as compared to the control group (Figure 5A,B). 

As a single agent, NER prevented BrM in three out of the five mice, whereas it had a limited effect on bone metastasis, and only one mouse was observed with no metastasis (Figure 5A,B). In contrast, CBZ as a single agent was more effective in decreasing BrM than lung, liver, and bone metastasis (Figure 5A,B). Overall, the data suggested that the combination of NER and CBZ effectively prevented metastasis to the brain with a partial effect on lung, bone, and liver metastasis. The differential response to combination therapy might be due to differential metastatic burden or due to poor response towards the therapy at different metastatic sites. 

## 3. Discussion

Targeted therapies, particularly the anti-ERBB2 antibody trastuzumab (Herceptin) and its derivatives, alone or in combination, have greatly improved survival in ERBB2^+^ BC patients [41,42,43]. In contrast, there are limited targeted therapies available with modest therapeutic responses to target distant metastasis in ERBB2^+^ BC patients [3,44,45]. The optimization of targeted therapies against metastatic ERBB2^+^ BC is challenging for several reasons, including molecular and metabolic adaptations in metastasizing cells, refractive organ-specific microenvironments, and lack of preclinical models to evaluate therapeutic targets [9,46,47,48]. In this study, we reported an orthotopic model of spontaneous ERBB2^+^ BC BrM that can be used to evaluate therapeutic approaches targeting both the primary tumor and distant metastases together, suggesting its high clinical relevance. Previously, an orthotopic mouse model of BrM using triple-negative cell lines MDA-MB-231 and 4T1 have been reported in NSG and BALB/c mice, respectively, which are suitable for therapeutic modalities against TNBC [49,50,51,52]. In the ERBB2^+^ BC subtype, both intracardiac and intracarotid models have been used for the evaluation of therapeutic modalities against BC BrM [24,25,53]. However, neither of these models are enough to understand the mechanisms of BC progression and metastasis, nor can they be used to test preventative therapies in BC. Recently, an ERBB2^+^ orthotopic model of spontaneous metastasis has been reported in BALB/c mouse [20]. However, the incidences of metastasis are more in lungs (~80%) as compared to bone (50%) and brain (60%). Conversely, the orthotopic nude mouse model described in this study is, physiologically, more relevant compared to intracardiac and intracarotid models and ideally suitable for the evaluation of therapeutic modalities against BC metastasis due to the presence of both matched primary and metastatic tumors. In addition, the SKBrM3^+^ orthotopic model exhibited consistently high incidences of metastasis to bone and brain, which recapitulates the clinical metastatic burden observed in metastatic BC patients [3]. For the assessment of metastasis in small animals like mouse models, BLI is the most commonly used method that is based on the luciferase activity of engineered metastatic cell lines [54,55]. Assessment of various organs derived from LUC+SKBrM3^+^ cell line implanted mice by BLI helped us to clearly identify the metastatic burden in different treatment groups. Therefore, BLI imaging of intact and freshly harvested tissues is an excellent approach to analyze incidences of metastasis using highly sensitive luciferin drugs and could be used to analyze metastases. However, further evaluation of molecular markers, radiological imaging, and histological analysis could be used for deeper insight of metastatic progression and associated pathways.

In line with previous reports that showed co-amplification of c-MET and ERBB pathways and their role in metastatic BC [24,56,57], we observed the higher expression of c-MET and ERBB1 receptors in ERBB2^+^ brain-seeking SKBrM3 cells as compared to its parental cell line, and in organoids derived from ERBB2-Tg mouse. As both the brain metastatic cell lines SKBrM3 and JIMT-1-BR3 showed altered molecular expression as compared to their parental cell line, our results support the existing notion that molecular expression is influenced during the gain of metastatic traits and in response to the microenvironment at metastatic sites [5,7,58]. For example, a recent multicenter study analyzed the genomic profile and subtype switching in BC patients who were diagnosed with BrM. Interestingly, there was differential genomic profiles in case of BrM as compared to their respective primary tumors and more than 20% patients showed subtype switching, including ERBB2+ patients [59]. In the case of BC, the amplification of ERBB receptors correlates with disease aggressiveness and distant metastasis [56,57]. Particularly, ERBB1 overexpression or its co-amplification with other ERBB receptors has been reported to favor distant metastasis [57]. Similarly, c-MET overexpression has been reported to contribute to BC metastasis, particularly to the brain. Previously, gene set enrichment analysis (GSEA) of a large cohort of BC patients (*n* = 710), 47 with BrM, showed a highly enriched c-MET-pathway in BrM patients [24]. In addition, the upregulation of c-MET is associated with BrM, not to the bone metastasis, and the knockdown of c-MET in brain-seeking cells has been reported to significantly increase BrM-free survival in vivo [24]. Interestingly, activation of c-MET was shown to induce high IL-1β secretion leading to an IL-8 and CXCL1 dependent feed-forward loop, creating a favorable environment for BrM. Further, the co-amplification and cross-talk between c-MET and ERBB1 pathways have been reported in different malignancies, including BC that regulates tumor progression, distant metastasis, and therapeutic resistance [29,30,60,61]. Previously, it has been reported that targeting both ERBB1 and c-MET receptors sensitizes cancer cells to targeted therapy [30]. Moreover, c-MET amplification has been reported to mediate resistance to ERBB1 inhibitors [61]. In another study, tissue samples from 825 BC patients were analyzed to correlate the expression of upregulated proteins. The study highlighted that ERBB1 overexpression correlated with high p-c-MET expression [62], substantiating our hypothesis that the co-targeting of ERBB1 and c-MET could be an important therapeutic strategy in the treatment of BC progression and metastasis. 

Our results in cell lines and organoids derived from the huERBB2-Tg mouse model provide evidence that combined inhibition of the ERBB1/ERBB2, and c-MET pathways could synergistically inhibit proliferation and metastasis in BC. Due to their intact tumor architecture, organoids are considered as a robust model for therapeutic drug testing in solid tumors, including BC [63,64,65,66]. The therapeutic effect of NER and CBZ in ERBB2^+^ organoids was further substantiated by in vivo findings in the orthotopic model of spontaneous metastasis. Both NER and CBZ have been reported to cross the BBB and, therefore, could be used to target BrM in our orthotopic model of spontaneous metastasis. Our results showed that the combination of NER and CBZ not only effectively decreased tumor volume but also prevented the incidences of metastasis in this BC orthotopic model. Previously, NER monotherapy in neoadjuvant settings has been shown to inhibit tumor growth and metastasis in the ERBB2^+^ orthotopic model of spontaneous metastasis [20]. However, the spontaneous metastasis was observed more in adrenal and lungs rather than bone and brain, which are the most common sites of metastasis in ERBB2^+^ BC patients. These studies suggest that interpretation of the therapeutic effect of NER monotherapy on distant metastasis might be difficult in the BALB/c model. In contrast, like our findings, the preventive effect of neoadjuvant NER therapy has been reported predominantly in case of BrM in the BALB/c model. However, the differential therapeutic response in different metastatic sites might be due to the altered molecular profile of metastatic cells and due to organ-specific microenvironments at different metastatic sites. Previous reports have provided solid evidence that cells metastasizing to different organs have different genetic and molecular profiles, leading to subtype switching; therefore, metastatic cells differ in their organotropism as well as their response to targeted therapies [7,58,59,67]. In addition, neoadjuvant NER therapy was reported less efficacious in preventing lung and liver metastasis, as compared to brain metastasis in the BALB/c model [20]. 

**Figure 6:**
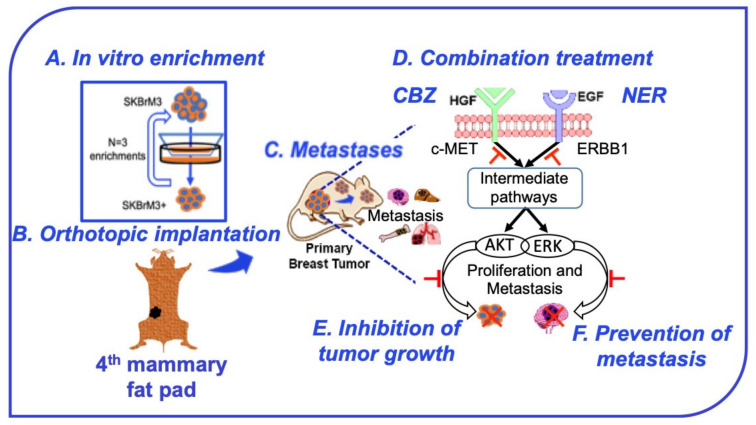
Schematic diagram showing targeted inhibition of the ERBB-c-MET axis to prevent BC metastasis in an orthotopic ERBB2^+^ mouse model. (**A**) In vitro enrichment of the SKBrM3 cell line using a Boyden chamber. (**B**) Orthotopic implantation of SKBrM3^+^ cells in fourth mammary fat pads of female nude mice. (**C**) Incidences of metastasis in distant organs including brain, bone, liver, and lung. (**D**) Combination treatment of NER and CBZ after 3 weeks of implantation. (**E**,**F**) Effect of combination therapy on primary tumor growth and metastasis.

Mechanistically, our findings show that combination therapy profoundly downregulated the MAPK/ERK pathway, which is downstream to c-MET and ERBB1 pathways. Previous studies have highlighted the MAPK/ERK pathway as a key mediator of proliferation and metastasis in metastatic BC that together with AKT/mTOR and STAT3 plays an essential role in progression of BrM [53,68,69]. A recent study showed that NER monotherapy inhibits proliferation of ERBB2^+^ BC via inhibition of ferroptosis [20]. In addition, other pathways, including PI3K, PARP, CDK4/6, FAK, etc., have been reported to play essential roles in BrM progression [70]. Therefore, combination therapies targeting multiple pathways might be an important strategy in the management of BrM in BC. We have summarized the treatment strategy in the graphical abstract (Figure 6). Although a three-week low dose regimen of NER and CBZ combination therapy decreased tumor growth and incidences of metastasis in our orthotopic model of spontaneous BC metastasis, we assume that treatment with the maximum tolerated dose in combination treatment would provide more profound in vivo efficacy. In addition, the orthotopic model of spontaneous metastasis will be a useful reagent to investigate underlying mechanisms involved in metastatic progression of BC and for evaluation of novel therapeutic approaches against BC metastasis.

## 4. Methods

### 4.1. Animal Use and Ethics

All animal work was performed as per the protocols (#17-019-04 FC) approved by the Institutional Animal Care and Use Committee (IACUC) at the University of Nebraska Medical Center (UNMC). The animals were kept in a specific pathogen-free animal care facility at UNMC. 

### 4.2. Cell Lines and Reagents

The SKBR3 cell line was procured from ATCC, and its metastatic derivative SKBrM3 was kindly gifted by Dr. Watabe from the Wake Forest School of Medicine, Winston Salem, North Carolina. Dr. Steeg from the National Cancer Institute kindly gifted us the JIMT-1-BR3 cell line, and the JIMT-1 cell line was obtained from Dr. Hamid Band at UNMC and maintained as described previously [71]. The drugs neratinib (Cat. No. S2150) and cabozantinib (Cat. No. S1119) used in the study were purchased from Selleckchem. 

### 4.3. Proliferation Assay 

Cell proliferation assays were performed, as described previously [72,73,74]. Briefly, five thousand cells per well were seeded in a 96-well plate in 10% FBS containing RPMI media for SKBR3 and SKBrM3 cell lines and in 10% FBS containing DMEM media for JIMT-1 and JIMT-1-BR3 cell lines. The next day, cells were starved in 2% FBS medium for 2 h prior to the drug treatments. After 2 h of serum starvation, cancer cells were treated with different concentrations of drugs, as mentioned in the Results Section. After incubation of cells at 37 °C for 48 h, we added 10 μL of 3-(4,5-dimethylthiazol-2-yl)-2,5-diphenyl-tetrazolium bromide (MTT, 5 mg/mL, Sigma, St. Louis, MO, USA) to each well. After adding MTT, plates were then incubated at 37 °C for 4 h. Absorbance was measured at 570 nm using a microplate reader (Spectramax 190 Microplate Reader, Molecular Devices, LLC, USA). 

### 4.4. Migration Assay

We performed the cell migration assay as published previously [75]; 1 × 10^6^ cells were seeded in 6-well plates in their respective media for 48 h in the presence of NER (1 μM) and CBZ (5 μM), and their combination. The drug-treated cells were trypsinized and seeded at a density of 5 × 10^5^ cells/well in a 6-well Boyden chamber in serum free media. In the lower chamber of 6-well plates, 10% serum containing media was added to demonstrate the in vitro effect of the above treatments on migration of brain metastasis and their respective primary cell lines. After 16 h, migrated cells on the opposite side of the trans-well were fixed in 100% methanol, stained with 0.1% crystal violet, imaged, and counted using an EVOS^®^ FL auto imaging system microscope CA, USA). 

### 4.5. Trans-Endothelial Migration

The trans-endothelial migration (TEM) was performed as described earlier [76]. Briefly, we seeded 5 × 10^5^ brain endothelial cells on the upper outer surface of the gelatin coated trans-well for 24 h. In the lower chamber, 5 × 10^5^ human astrocytes were cultured for 24 h. the next day, drug treated brain tropic SKBrM3 cells were seeded onto the top of endothelial cells in a trans-well insert. All the three cells types were incubated for the next 16 h in co-culture conditions, and transmigrated cells were counted by fixing cells in 100% methanol and stained with 0.1% crystal violet. Transmigrated cells were imaged and counted using an EVOS^®^ FL auto imaging system microscope (Life Technologies, CA, USA).

### 4.6. Immunohistochemistry 

Immunohistochemistry (IHC) was performed on tumor tissues as described earlier [77]. Briefly, tissue sections were kept overnight at 58 °C and followed by xylene wash and alcohol gradient-based hydration. Citrate buffer (pH = 6) was used for antigen retrieval, followed by peroxidase quenching using 0.3% H_2_O_2_ in methanol. Following 3 washes in water, tissues were blocked in 2.5% horse serum and incubated overnight in primary antibody cocktail, ERBB1 (D38B1; 1:400), ERBB2 (Cat. No. 2242; 1:200), and c-MET (Cat. No. 4560; 1:200, purchased from Cell Signaling technologies, Beverly, MA, USA. The next day, after washing off unbound primary antibodies, an HRP-conjugated antibody cocktail (ImmPress Universal antibody kit, Vector Laboratories, Burlingame, CA, USA) was used for 30 min at RT and developed using 3-3’ diaminobenzidine solution (DAB substrate kit (SK-400), Vector Laboratories, Burlingame, CA, USA. Following counterstain with hematoxylin, the slides were dehydrated in an alcohol gradient from 25% to 100%, followed by 3 xylene washes. After that, air-dried tissue sections were mounted using paramount media (Fisher Scientific, PA, USA) and visualized under the microscope.

### 4.7. Immunofluorescence 

For immunofluorescence in cell lines and tissues, we followed previously published protocol with slight modifications [78,79]. Briefly, the coverslip adhered cells were fixed in 4% paraformaldehyde for 15 min at RT, washed in 0.1% glycine, and permeablized in 0.1% TritonX100 for 10 min at RT. After washing in PBS, cells were blocked in 10% normal goat serum for 2 h, followed by incubation in primary antibodies specific to ERBB1 (D38B1; 1:400), ERBB2 (Cat. No. 2242; 1:200), and c-MET (Cat. No. 4560; 1:200) for O/N incubation in a humidified chamber. The next day, slides were washed 3 times in PBS containing 0.1% Tween-20 (PBS-T) and incubated for 45 min at RT with fluorophore-conjugated secondary antibodies. Following four washes in PBS-T, the cells or tissue sections were mounted in DAPI-containing mounting media and then visualized under the microscope. 

### 4.8. Western Blotting 

Immunoblotting was performed as described previously [80]. Briefly, 40 μg of each cell lysate was separated by 10% SDS–PAGE and then transferred to a polyvinylidene fluoride (PVDF) membrane (Millipore, USA). The membranes were blocked in 5% skimmed milk for 1 h and incubated overnight at 4 °C with primary antibodies against pERBB1 (D7A5:1:1000), CST; ERBB1 (D38B1; 1:1000), CST; p-c-MET (D26; 1:1000), CST; c-MET (4560; 1:1000), CST; pERBB2 (2247s; 1:1000), CST; ERBB2 (2242; 1:1000), CST; pERK (9102; 1:1000), CST; ERK (9101; 1:1000), CST; pAKT (9271; 1:1000), CST; AKT (4691; 1:1000), CST. All these antibodies were purchased from Cell Signaling technologies, Beverly, MA, USA, unless, mentioned specifically. Following washes with PBST, the membranes were incubation with HRP-conjugated anti-rabbit or anti-mouse IgG (1:3000; Santa Cruz Biotechnology, Dallas, Texas, USA) for 1 h. The proteins were visualized using ECL-plus reagents (Amersham Biosciences Corp., USA). 

### 4.9. Organoid Culture and Treatment Assay

Tumor tissues were enzymatically digested and processed as described previously [81]. Briefly, freshly harvested tissues were digested with 0.012% (*w/v*) collagenase XI (Sigma) and 0.012% (*w/v*) dispase (GIBCO, MD, USA) in DMEM media containing 1% FBS (GIBCO) and embedded in growth factor reduced Matrigel (BD Biosciences, San Jose, CA, USA). The organoids were cultured in AdDMEM/F12 (GIBCO) media supplemented with 0.1% insulin–transferrin–selenium (ITS-G) (100×) (Gibco™, MD, USA), FGF10, and FGF 2 (PreproTech, Cranbury, NJ, USA), in a 5% CO_2_ incubator at 37 °C. On the 5th day, the tumor organoids were treated with drugs, and changes in the size and morphology were followed consecutively for 7 days. The bright-field images were acquired on an EVOS^®^ FL auto imaging system microscope (Life Technologies, CA, USA), followed by quantification of the change in area of selected organoids.

### 4.10. Orthotopic Mouse Model

SKBrM3 cells were harvested at 70–80% confluency using 0.025% trypsin and seeded at 5 × 10^5^-cells/well density in the upper chamber of a 0.8-μm pore size trans-well inserted in 6-well plates containing 1.5 mL media. The migrated cells were collected from the wells and then subjected to two more cycles of enrichment. After analyzing the luciferase activity, 1 × 10^6^ enriched SKBrM3^+^ (or BrM3^+^) cells in 100 μL of PBS were orthotopically implanted in the fourth mammary fat pad of 6- to 8-week-old female nude mice. Tumor growth was followed by caliper-based measurements, as well as by imaging the mice on a small-animal in vivo imaging system (IVIS) on a weekly basis. 

### 4.11. Drug Treatment and Animal Imaging

We evaluated the effect of oral doses of NER (20 mg/kg/mouse) alone, CBZ (20 mg/kg/mouse) alone, or their combination in our orthotopic model of ERBB2^+^ spontaneous BC metastasis using 6- to 8-week-old female nude mice. The tumor volume and body weight of each mouse were measured every 4th or 5th day. Post-treatment, the mice were first injected intraperitoneally (IP) with 15 mg/kg bodyweight of D-luciferin (Cat No. 122799, Perkin Elmer, Akron, OH, USA), and imaging was performed using small animal IVIS to analyze the effect on tumor growth. Further, the mice were sacrificed as per the IACUC guidelines and organs associated with BC distant metastasis including bone (from hind limbs), brain, liver, and lungs were harvested for single organ imaging to investigate the micrometastases. Following imaging and weighing of the tumors, the organs were preserved in 10% buffered formalin for fixation and further analysis.

### 4.12. Statistical Analysis

The statistical analysis was performed using Student’s *t*-test (* *p* < 0.05; ** *p* < 0.01; *** *p* < 0.001). For comparison among the independent treatment groups, we used a one-way analysis of variance (ANOVA) with a cutoff of *p* < 0.05. Tukey’s multiple comparison test was performed to analyze the statistical significance for difference in tumor weights as well as tumor volumes among different treatment groups using two-way ANOVA with * *p* < 0.05; ** *p* < 0.01; *** *p* < 0.001; and **** *p* < 0.0001.

## 5. Conclusions

We report in this paper an orthotopic mouse model for spontaneous BC metastasis that shows high incidences of BrM along with other clinically relevant distant organ metastases, including bone, liver, and lungs. Next, in concordance with high expression of ERBB1 and c-MET receptors, the combination treatment of NER and CBZ showed significant anti-proliferative effects in brain seeking cell line SKBrM3 and ERBB2^+^ organoids and elicited a profound inhibitory effect on cell migration in vitro. Our in vitro data was further substantiated by an in vivo preventive treatment approach where the combination treatment with NER and CBZ significantly reduced the primary tumor burden and demonstrated preventive effects on metastatic progression to the brain in an orthotopic model of spontaneous BC metastasis (Figure 6). Overall, the combination of NER and CBZ could effectively inhibit BrM and targeting the c-MET/ERBB1 axis could be a unique strategy for preventing ERBB2^+^ BC BrM.

## Supplementary Materials

The following are available online at https://www.mdpi.com/2072-6694/12/10/2838/s1, Figure S1: Expression of ERBB1, ERBB2, and c-MET in brain seeking and their parental cell lines, Figure S2: Expression of ERBB1, ERBB2, and c-MET in brain seeking and their parental cell lines, Figure S3: Effect of NER (1 μM), CBZ (5 μM), and their combination on SKBR3 and SKBrM3 cell lines in vitro, Figure S4: Effect of NER (1 μM), CBZ (5 μM), and their combination on JIMT-1 and JIMT-1-BR3 cell lines in vitro, Figure S5: WB analysis to compare SKBrM3 and Boyden chamber enriched SKBrM3+ cell line, with an analysis of ERBB1, ERBB2, c-MET, vimentin, and ZEB1. Corresponding β-actin blot was shown as loading control.
