# Supplementary material for: Blocking c-MET/ERBB1 Axis Prevents Brain Metastasis in ERBB2+ Breast Cancer
Source: Cancers (Basel). 2020 Oct 1;12(10):2838. doi: 10.3390/cancers12102838 (PMC7601177; doi:10.3390/cancers12102838)
Supplement: Supplementary file 1 [file cancers-12-02838-s001.pdf]

cancers

MDPI

Supplementary Figures

Blocking c-MET/ERBB1 Axis Prevents Brain Metastasis in ERBB2+ Breast Cancer

Shailendra K Gautam, Ranjana K Kanchan, Jawed A Siddiqui, Shailendra K Maurya, Sanchita Rauth, Naveenkumar Perumal, Pranita Atri, Ramakanth C Venkata, Kavita Mallya, Sameer Mirza, Moorthy P Ponnusamy, Vimla Band, Sidharth Mahapatra, Maneesh Jain, Surinder K Batra and Mohd Wasim Nasser

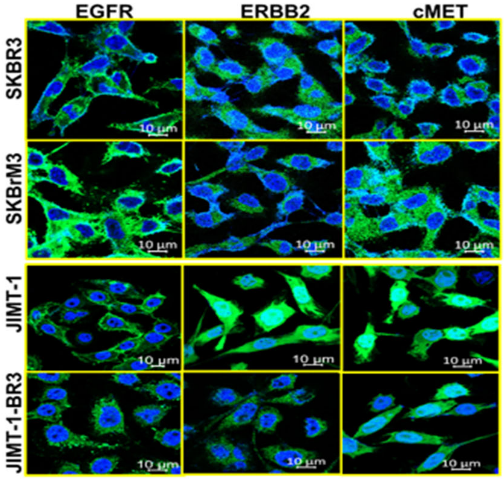

Figure S1B

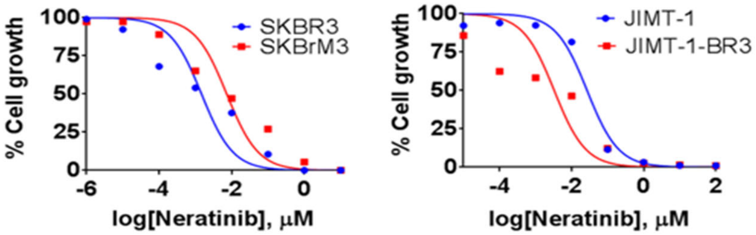

Figure S1. Expression of ERBB1, ERBB2, and c-MET in brain seeking and their parental cell lines. (**A**). Expression of ERBB1, ERBB2, and c-MET in brain seeking and their parental cell lines. Immunofluorescence assay to show the expression of ERBB1, ERBB2, and c-MET in brain seeking cells SKBrM3 and JIMT-1-BR3 and their respective parental BC cell lines. X-axis shows the target molecule and Y-axis shows the cell line. For imaging, we used 60 X magnification in Zeiss LSM confocal microscope (LSM800) and images are presented at magnification of 10 µm. (**B**) Dose response curve of NER in brain metastatic and their parental cell lines. Dose response curve of neratinib (NER) in SKBrM3 and JIMT-1-BR3 cells and their respective parental cell lines SKBR3 and JIMT-1 using MTT assay. X-axis represents log scale showing increasing concentration of NER, whereas Y-axis shows effect of NER on percent cell growth.

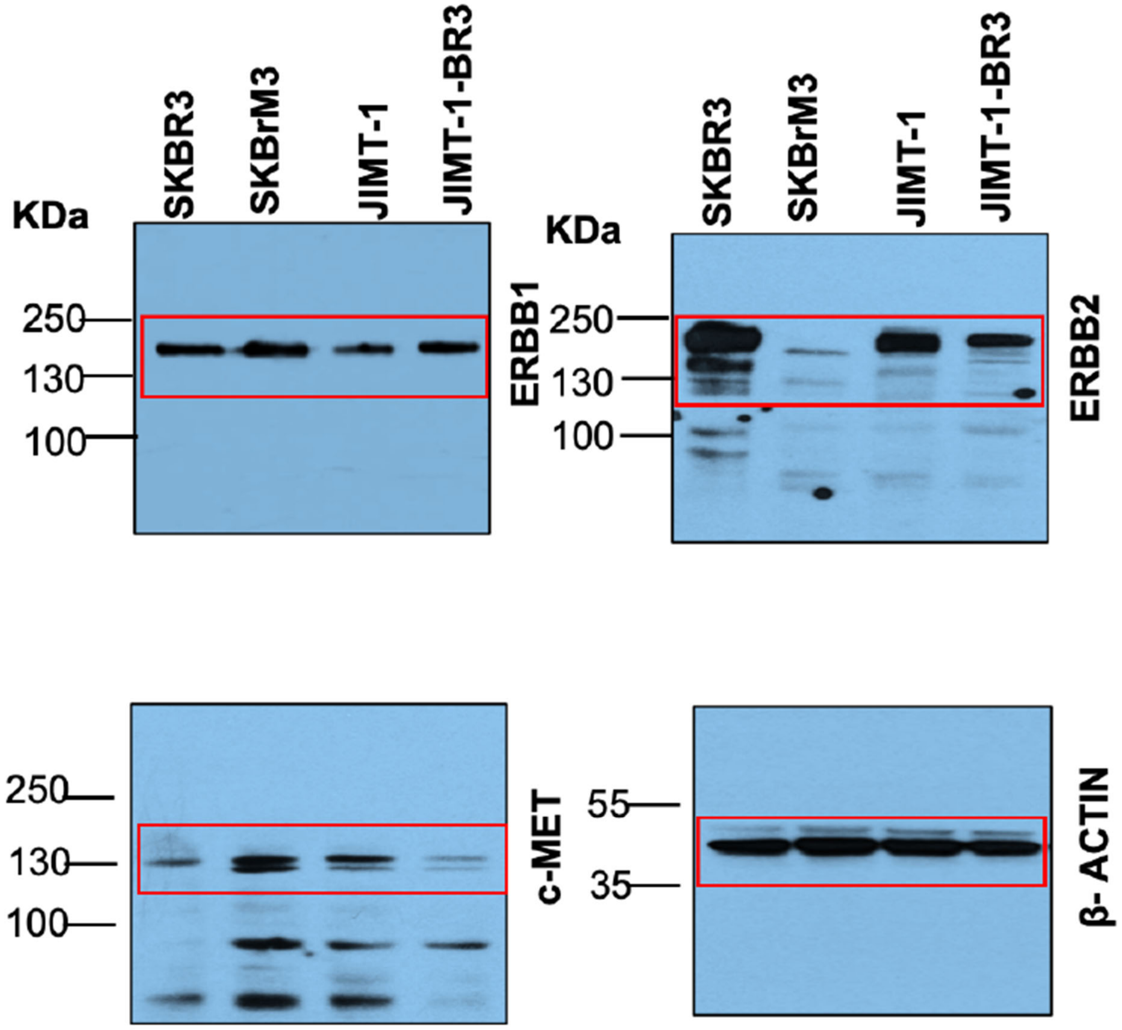

Figure S2. Expression of ERBB1, ERBB2, and c-MET in brain seeking and their parental cell lines (Figure 1A).

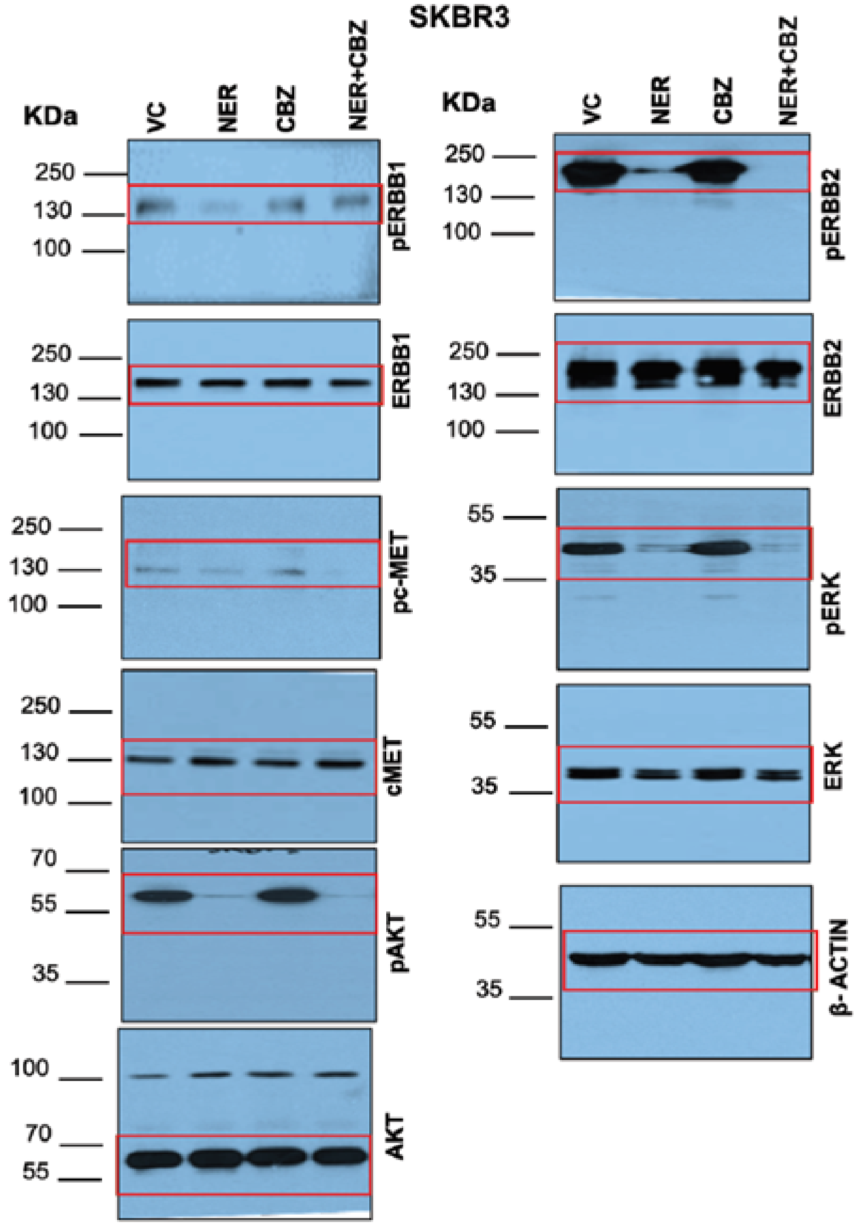

Figure S3. Effect of NER (1 μM), CBZ (5 μM), and their combination on SKBR3 and SKBrM3 cell lines in vitro (Figure 2C).

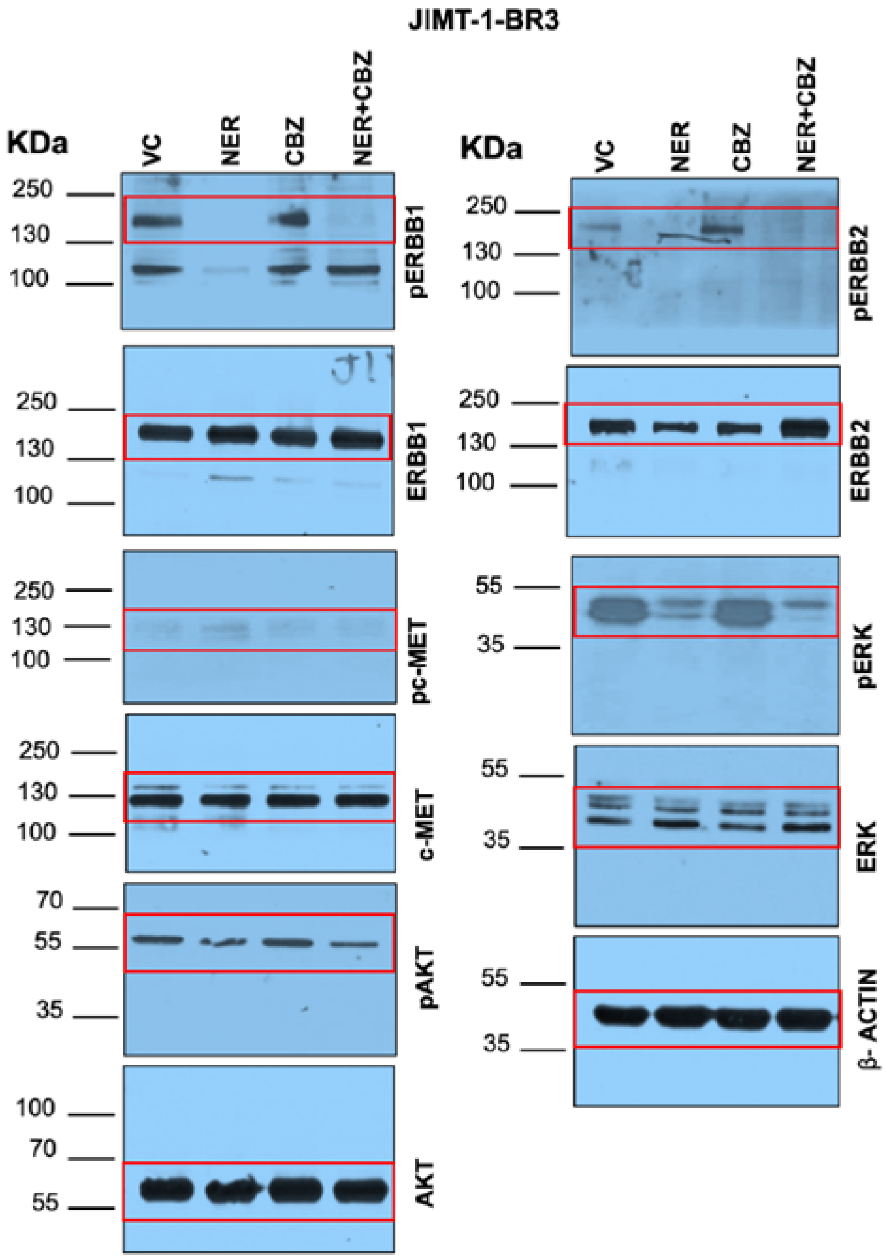

Figure S4. Effect of NER (1 μM), CBZ (5 μM), and their combination on JIMT-1 and JIMT-1-BR3 cell lines in vitro (Figure 2D).

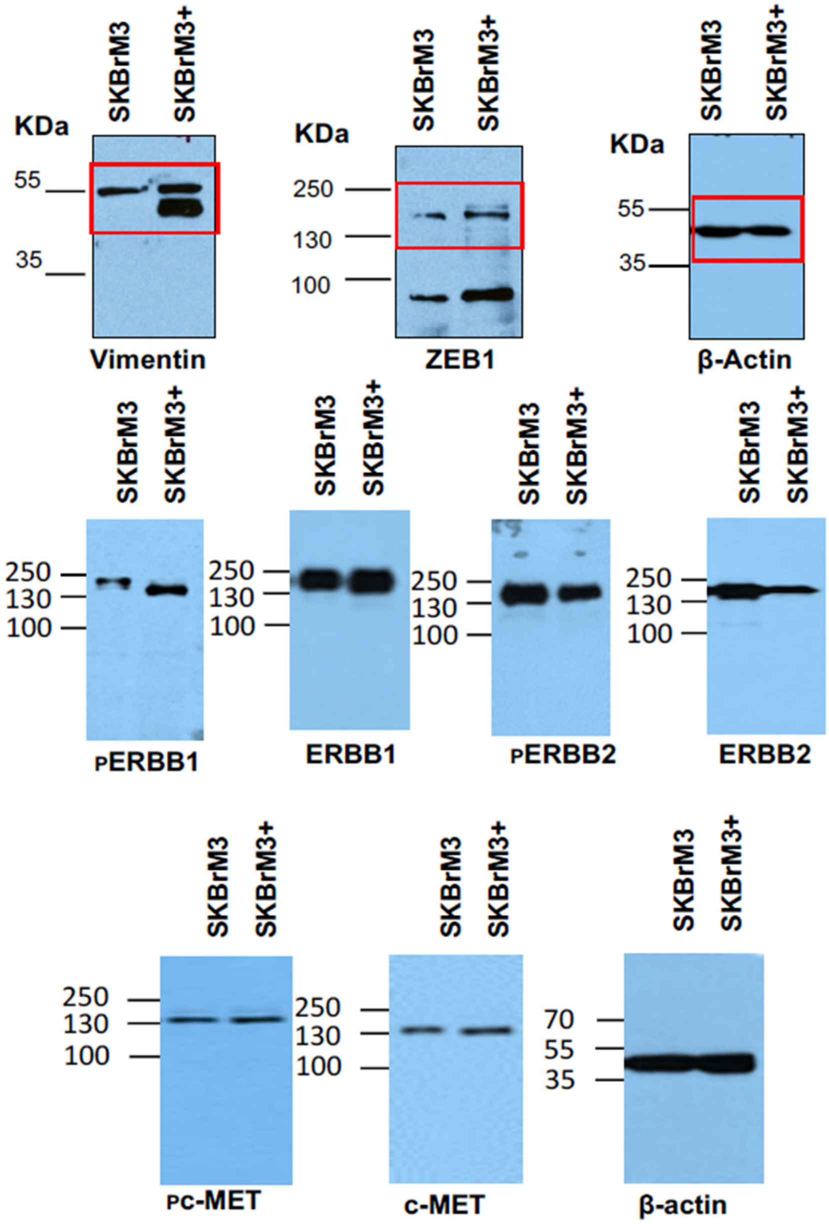

Figure S5. WB analysis to compare SKBrM3 and Boyden chamber enriched SKBrM3+ cell line, with an analysis of ERBB1, ERBB2, c-MET, vimentin, and ZEB1. Corresponding β-actin blot was shown as loading control (Figure 3B).
